# Circumferential shoulder laceration after posterior axilla sling traction: a case report of severe shoulder dystocia

**DOI:** 10.1186/s12884-020-03526-2

**Published:** 2021-01-11

**Authors:** Allison R. McCarter, Regan N. Theiler, Enid Y. Rivera-Chiauzzi

**Affiliations:** Department of Obstetrics and Gynecology, Mayo Clinic, 200 First St SW, 55905 Rochester, MN USA

**Keywords:** Case report, Complication, PAST, PAST technique, Shoulder dystocia, Posterior axillary sling traction

## Abstract

**Background:**

Shoulder dystocia is an unpredictable and potentially catastrophic complication of vertex vaginal delivery. Posterior axilla sling traction (PAST) has recently been proposed as a method to resolve severe shoulder dystocia when commonly used techniques have failed.

**Case presentation:**

A 33-year-old woman (gravida 5, para 0) at 35 weeks, 1 day gestation underwent induction of labor for poorly controlled type 2 diabetes mellitus. Delivery of the large-for-gestational-age infant (4,060 g) was complicated by intractable shoulder dystocia, relieved at 3 minutes with PAST, resulting in a deep, circumferential laceration of the fetal posterior shoulder and contralateral phrenic nerve palsy.

**Conclusions:**

PAST provides a potentially lifesaving option during intractable shoulder dystocia. Simulation or education about the technique facilitates its use when standard maneuvers fail. It is important to disseminate information about potential complications associated with these novel maneuvers.

## Background

Shoulder dystocia is an often unpredictable and potentially catastrophic obstetric emergency in which the descent and delivery of the fetal shoulders are obstructed by the bony pelvis. It is typically diagnosed by failure of the shoulder to descend upon application of gentle axial traction to the fetal head [1]. Shoulder dystocia is thought to complicate 0.2–3% of vertex deliveries [2], and risk factors include preexisting or gestational diabetes mellitus, maternal obesity, macrosomia, and late-term gestation. However, in most cases, shoulder dystocia occurs in the absence of identifiable risk factors [3, 4].

Shoulder dystocia is associated with a multitude of adverse maternal and fetal outcomes. Maternal complications may include anal sphincter injury and postpartum hemorrhage, and neonatal complications include brachial plexus palsy, fracture of the fetal humerus or clavicle, hypoxic ischemic encephalopathy (HIE), and even neonatal death [5]. Initial maneuvers to alleviate shoulder dystocia, including McRoberts positioning and application of suprapubic pressure, result in resolution of shoulder dystocia in up to 58% of cases [6, 7]. Several internal maneuvers have been described to alleviate persistent shoulder dystocia, including delivery of the posterior arm and shoulder, and rotational techniques (Wood and Rubin maneuvers). Advanced measures for intractable shoulder dystocia include replacement of the fetal head with emergent cesarean delivery (Zavanelli maneuver), abdominal rescue, symphysiotomy, and intentional clavicle fracture. Longer duration of intractable shoulder dystocia and a higher number of attempted maneuvers are associated with worsening adverse maternal and neonatal outcomes [5, 7].

In 2009, Hofmeyr and Cluver [8] described the posterior axilla sling traction (PAST) technique as a new method of relieving severe shoulder dystocia. The sling, created from a suction catheter or a firm urinary catheter, is placed through the fetal posterior axilla and put on traction. The technique is intended to tilt the fetal shoulders or rotate the neonate to relieve the obstruction and facilitate delivery in the setting of severe shoulder dystocia [9]. Here, we describe a novel complication associated with successful application of the PAST technique. The reporting of this case is in compliance with the CARE guidelines [10].

## Case presentation

A 33-year-old woman (gravida 5, para 0) was referred to maternal-fetal medicine specialists at Mayo Clinic for poorly controlled type 2 diabetes mellitus. She was at 35 weeks and 0 days gestation, as determined by her last menstrual period (and consistent with the 6-week ultrasound). Her insulin requirements drastically increased throughout the course of pregnancy and were in excess of 1,200 units daily at the time of consultation. The pregnancy was additionally complicated by pre-eclampsia without severe features diagnosed at 33 weeks’ gestation, maternal obesity (body mass index, 47 kg/m^2^), excess weight gain (16.5 kg), obstructive sleep apnea, and hypothyroidism. A fetal growth ultrasound estimated the fetal weight as 3,870 g. Given her worsening diabetes in the setting of multiple comorbid conditions, induction of labor the following day (35 weeks, 1 day gestation) was recommended [11].

Induction was initiated with misoprostol and a Foley bulb and was augmented by oxytocin and artificial rupture of membranes. Labor progressed normally, based on Consortium on Safe Labor guidelines, with the active phase starting at 6 cm [12]. The active phase of labor lasted 4 hours and 5 minutes, with cervical dilation at a rate of 1 cm/hour. Fetal station throughout the active phase of labor remained unchanged at 0 station. The fetal heart rate tracing was category I throughout active labor. She pushed for 2 hours and 8 minutes, with appropriate descent of the fetal vertex. The fetal heart rate tracing was category II, with moderate variability, variable decelerations, and rapid return to baseline while pushing. The fetal head was delivered in the occiput anterior position without asynclitism and required manual restitution to the left occiput transverse position. The fetal shoulders failed to deliver with gentle downward traction to the fetal head.

A shoulder dystocia was diagnosed 20 seconds after delivery of the fetal head and additional providers were called to the delivery room. The fetal shoulders had traversed the pelvic inlet in the anteroposterior position at the diagnosis of shoulder dystocia. Initial maneuvers, including suprapubic pressure and McRoberts positioning, were unsuccessful at releasing the anterior shoulder. A right mediolateral episiotomy was cut to create space, followed by attempted delivery of the posterior arm. Rotational techniques were then used, with the Rubin maneuver followed by the Wood corkscrew, resulting in clockwise rotation of the fetus to a 60° oblique angle. The Gaskin maneuver was not attempted because of dense neuraxial analgesia. Replacement of the fetal head with emergent cesarean delivery (Zavanelli maneuver), abdominal rescue, and symphysiotomy were not rapidly feasible with the maternal body habitus. Repeated attempts to deliver the posterior arm were unsuccessful, as was attempted fracture of the fetal clavicle. A second faculty obstetrics physician came to assist after approximately 2 minutes of dystocia time, and another attempt at the above-described maneuvers was again unsuccessful.

At approximately 2 minutes and 20 seconds of dystocia time, a 16 French latex urinary catheter was positioned below the posterior fetal shoulder, per the PAST technique [8]. Downward traction on the axillary sling was initially unsuccessful in releasing the posterior arm, but it allowed for rotation to access the posterior arm for another attempt at delivery. Downward traction was again applied to the axilla sling, resulting in delivery of the posterior arm after approximately 3 minutes of dystocia time. The anterior shoulder and trunk required additional rotation and traction to facilitate delivery of the fetal body. In total, the fetus was delivered 4 minutes after delivery of the fetal head.

Delivery was further complicated by a retained placenta that required manual extraction, as well as by postpartum hemorrhage due to uterine atony. Uterotonic agents, including oxytocin, carboprost, and misoprostol, were administered. Tranexamic acid was administered before placement of a Bakri uterine balloon with successful tamponade. The quantitative blood loss was 1,640 mL.

**Fig. 1:**
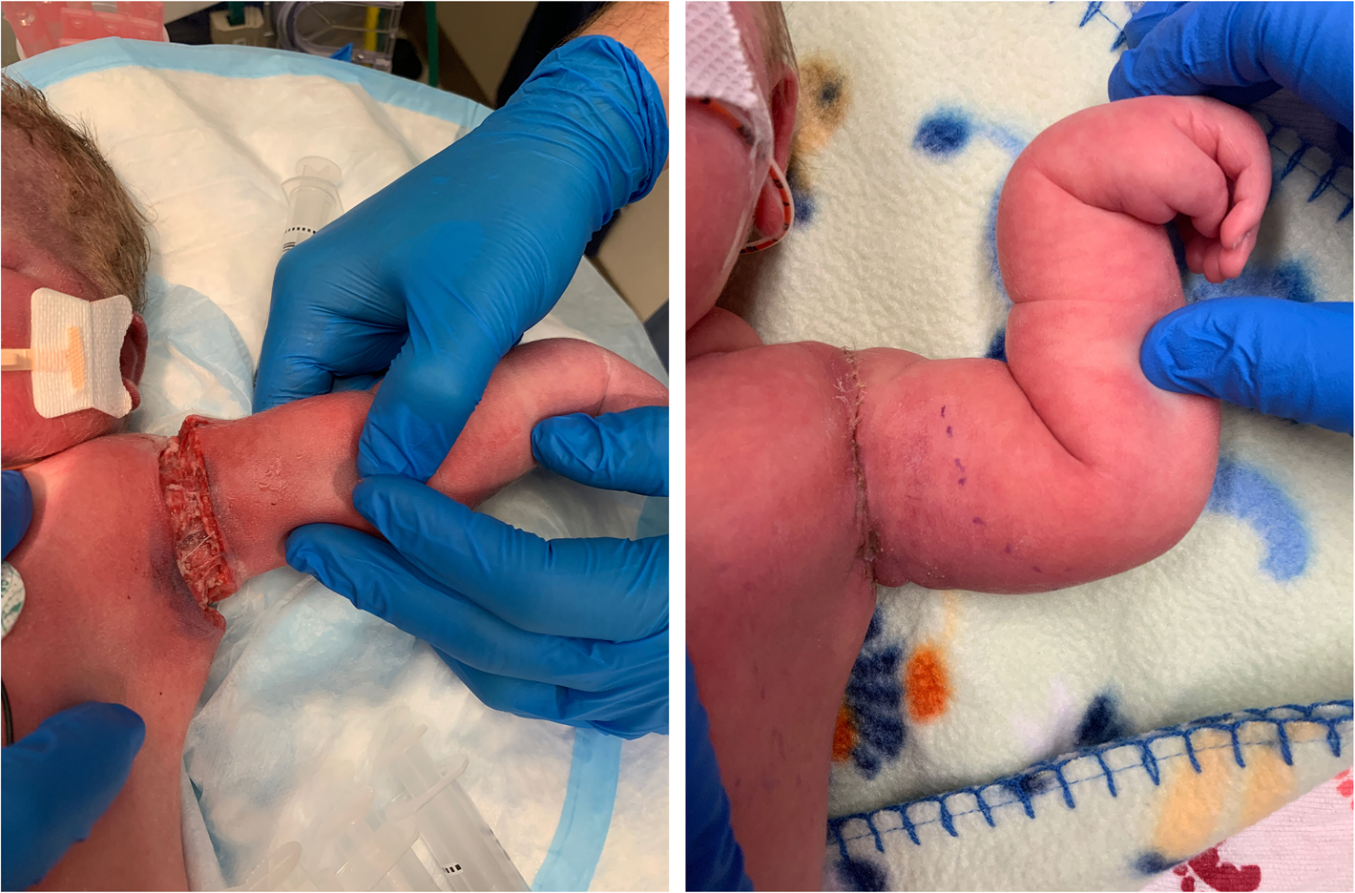
Left, Circumferential laceration of the posterior arm (day of birth) attributable to posterior axilla sling traction with a urinary catheter for severe shoulder dystocia. Right, Progression of healing (28th day of life) after definitive wound closure 2 weeks prior

**Fig. 2:**
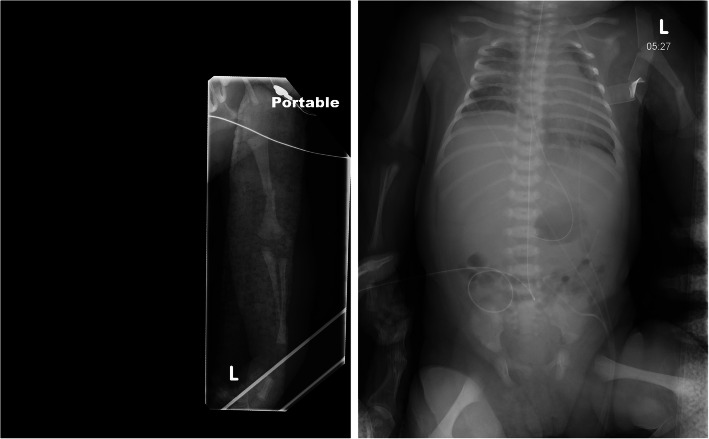
Left, Left midshaft humerus fracture on day of birth. Extension of the elbow and flexion and pronation of the wrist are evident and characteristic of Erb palsy. Right, Radiograph shows an elevated right hemidiaphragm, attributable to contralateral (right-sided) phrenic nerve injury

At delivery, the infant was apneic and limp, weighing 4,060 g. Initial neonatal stabilization with cardiopulmonary resuscitation was immediately required. Apgar scores were 0, 4, and 5 at 1, 5, and 10 minutes, respectively. Arterial cord gas showed a pH of 7.12, HCO_2_ of 25 mmol/L, and base excess of − 3 mmol/L. At 15 minutes of life, venous blood gas showed a pH of 6.78, pCO_2_ of 29 mm Hg, and a base excess of − 30 mmol/L, consistent with metabolic acidosis attributable to lactic acid accumulation from cardiopulmonary arrest. A large, circumferential laceration was noted on the left (posterior) shoulder of the neonate (Fig. 1). After initial neonatal stabilization, a therapeutic hypothermia protocol was initiated. Initial electroencephalographic recordings showed slowing consistent with encephalopathy, but the neonate continued to improve, with no seizure activity noted throughout 5 days of continuous monitoring. Magnetic resonance imaging on the fifth day of life did not show diffusion restriction suggestive of anoxic brain injury. Left-sided (posterior) Erb palsy was clinically evident, but electromyography showed normal median, radial, and ulnar nerve stimulation. Injury to the right (anterior) phrenic nerve was diagnosed by intermittent paralysis of the right hemidiaphragm (Fig. 2). The infant remained intubated for 13 days because of respiratory failure of the newborn and hemidiaphragm paralysis. The circumferential left shoulder laceration required 3 bedside débridement and irrigation procedures, and the wound was surgically closed on the twelfth day of life. The left humeral fracture was managed conservatively with immobilization. At 1 year of life, the neonate had full function and range of motion of the bilateral upper extremities. The left shoulder laceration had healed appropriately, although it may require lengthening to allow for future growth of the extremity.

## Discussion and conclusions

The PAST technique was initially described as a method to alleviate intractable shoulder dystocia in the setting of fetal demise [13]. It has subsequently been used in live births complicated by shoulder dystocia when delivery of the posterior arm and rotational maneuvers have failed. A recent review evaluated all published cases of PAST for severe shoulder dystocia and noted a 94.7% success rate for delivery of the fetus [14]. Fracture of the posterior humerus occurred in 15.8% and brachial plexus palsy of the anterior arm was noted in 26.3% of these deliveries with PAST.

The duration of shoulder dystocia is not an accurate predictor of neonatal asphyxia. In a series reviewing cases of shoulder dystocia [5], the mean time from head to body delivery for patients with HIE was 10.75 minutes (range, 3–20 minutes), and 47% of neonatal deaths occurred with a head-to-delivery time of less than 5 minutes. Further, deliveries with a high number of obstetric maneuvers (> 5) were associated with HIE.

Despite the number of published cases describing the PAST maneuver, we are unaware of any reported cases with a laceration injury to the posterior arm. The mechanism of injury was thought to be a shearing injury because, as traction was applied to the sling, the latex urinary catheter stretched. The ideal instrument to use with the PAST procedure has not been established, but plastic suction or intravenous tubes have been described in the literature [13]. In this case, a urinary catheter was used because it was immediately available to delivering providers. At the point in which the urinary catheter was used, alternative measures for intractable shoulder dystocia, including replacement of the fetal head with emergent cesarean delivery (Zavanelli maneuver), abdominal rescue, and symphysiotomy were deemed a greater risk than attempting the PAST procedure. We have advocated for our institution to provide ready access to neonatal suction catheter tubing for use with the PAST technique for subsequent cases of intractable shoulder dystocia.

Until the aforementioned case, the PAST procedure had never been performed at our institution, although some providers had read about it or had been taught the technique during simulation training. Our success with this delivery reinforces the importance of having multiple options to manage the rare occurrence of intractable shoulder dystocia [14].

It is important to disseminate information about the potential complications, such as circumferential laceration of the fetal shoulder, that are associated with these novel maneuvers. The impact of this complication was moderately severe and required 3 bedside débridement and irrigation procedures before wound closure. Our case was also complicated by concurrent posterior shoulder brachial plexus injury with contralateral phrenic nerve injury, an uncommon complication of difficult deliveries. To our knowledge, contralateral phrenic nerve injury with Erb palsy at birth is rare [15], although ipsilateral phrenic nerve injury is well described [16, 17]. At 1 year of life, the child had no known residual neurologic or musculoskeletal impairment. As shown by this case, PAST provides another potentially life-saving option for intractable shoulder dystocia, and simulation or education about the technique facilitates its use when standard maneuvers have failed.

## Data Availability

Not applicable.

## Abbreviations

HIE: Hypoxic ischemic encephalopathy
PAST: Posterior axilla sling traction
